# Non-syndromic Cleft Lip and Palate Polymorphisms Affect Normal Lip Morphology

**DOI:** 10.3389/fgene.2018.00413

**Published:** 2018-10-24

**Authors:** Caryl Wilson-Nagrani, Stephen Richmond, Lavinia Paternoster

**Affiliations:** ^1^Department of Orthodontics, University Dental Hospital, Cardiff, United Kingdom; ^2^MRC Integrative Epidemiology Unit, School of Social and Community Medicine, University of Bristol, Bristol, United Kingdom

**Keywords:** lip morphology, ALSPAC, cleft, palate, skeletal pattern, craniofacial, NOG, facial

## Abstract

Non-syndromic cleft lip with or without palate (NSCL/P) is a frequent malformation of the facial region. Genetic variants (SNPs) within nineteen loci have been previously associated with NSCL/P in GWAS studies of European individuals. These common variant SNPs may have subtler effects on the morphology of the lip and face in unaffected individuals. Several studies have investigated the genetic influences on facial morphology using land-marking methods, but these landmarks are sparse in the lip region. The aim of this study is to assess for associations between the nineteen NSCL/P SNPs and normal lip phenotypes, using a detailed categorical scale. Three-dimensional laser scanned facial images were obtained of 4,747 subjects recruited from the Avon Longitudinal Study of Parents and Children (ALSPAC) and genetic data was available for 3,643 of them. A polygenetic risk score (PRS) combining the nineteen NSCL/P SNPs was associated with V-shaped Cupid's bow (*P* = 3 × 10^−4^) and narrow philtrum (*P* = 2 × 10^−4^) phenotypes. Analysis of individual SNPs found strong evidence for association between rs227731 and skeletal II pattern (*P* = 5 × 10^−6^). This study finds that known NSCL/P SNPs affect lip phenotypes in the general population, and an increased PRS is associated with narrow philtrum and V-shaped Cupid's bow. However, the difference in NSCL/P PRS between people with and without certain lip features is unlikely to be great enough to serve as a useful marker of NSCL/P risk.

## Introduction

Cleft lip with or without palate (CL/P) is the most common craniofacial disorder worldwide. It has varying prevalence ranging from approximately 1 in 500 to 1 in 1,000 live births depending on ethnicity and country (Mossey et al., 2009; Klotz et al., 2010). Cleft palate only (CPO) is considered as a separate entity to cleft lip and/or palate, and is thought to have a distinct etiology (Mossey et al., 2009).

The corrective treatment of CL/P requires extensive multi-disciplinary management, and poses an enormous burden on not only upon those affected, but also their families and social and health care systems (Mossey et al., 2009). Therefore, there is significant interest in identifying non-clefting phenotypic markers that might be used for risk prediction in the general population, which would benefit the development of prevention strategies.

There are two main subtypes: syndromic and non-syndromic CL/P. Syndromic cleft lip/palate (SCL/P) accounts for around 30% of cases, and presents with additional characteristic features, NSCL/P have no other observed defects. SCL/P can be categorized according to chromosomal abnormalities, Mendelian single gene syndromes, teratogenic effects or unknown syndromes. It is due to these modes of inheritance that SCL/P have high familial aggregation rates (Dixon et al., 2011).

NSCL/P comprise the majority of clefts, and these arise sporadically, with modest recurrence rates. The etiology is hypothesized to be multifactorial and polygenic (Cobourne, 2004), with many genetic variants, as well as environmental factors, such as smoking (Little et al., 2004; Beaty et al., 2013), alcohol (Honein et al., 2007; Romitti et al., 2007) and certain medications or nutrient deficiencies suggested to play a role (Park-Wyllie et al., 2000; Mitchell et al., 2003).

Many variants across several loci have been associated with NSCL/P in populations of various ancestries (Ingersoll et al., 2010; Butali et al., 2014; de Araujo et al., 2016; Leslie et al., 2016; Jia et al., 2017; Yu et al., 2017). Nineteen of these independent genetic variants have been associated with NSCL/P in genome-wide association studies (GWAS) of European populations. Eleven of these SNPs; rs560426, rs742071, rs861020, rs7590268, rs12543318, rs987525, rs7078160, rs8001641, rs1880646, rs227731 and rs13041247, have achieved subsequent replication in further studies (Ludwig et al., 2012; Mangold et al., 2012; Beaty et al., 2013; Jia et al., 2015).

Several research attempts have been made previously using GWAS to determine how genetic variants affect facial morphology; these have used traditional phenotyping methods, which involve land-marking regions of the face, and subsequent analysis of distances, or principal component analysis (Liu et al., 2012; Paternoster et al., 2012; Peng et al., 2013; Shaffer et al., 2016). The majority of the successes so far have involved regions attributable to bony landmarks, in particular those that have good reproducibility (Peng et al., 2013). More recently, a data driven approach of phenotyping has been described, which successfully identified loci in cranial neural crest cells (Claes et al., 2018).

Thus, the results of these studies have demonstrated that a handful of common variants which contribute to normal variation in human facial morphology, lie within genetic regions known to contribute to craniofacial development or syndromes.

A few studies have suggested that relatives of affected NSCL/P children may have altered facial phenotypes, compared to those with no family history of clefting (controls). These alterations can range from defects in the orbicularis oris muscle (Neiswanger et al., 2007), alterations in dental anomalies or malocclusions (Prochazkova and Tolarova, 1986), to variations in craniofacial skeletal disproportions (Mossey et al., 1997, 2010; McIntyre and Mossey, 2004).

As such, a few studies have explored how specific NSCL/P SNPs may affect normal facial morphology (Boehringer et al., 2011; Miller et al., 2014; Howe et al., 2018). These found that unaffected relatives of individuals with NSCL/P had variations in facial proportions in the A-P and transverse distances, as well as relative facial asymmetry compared to controls (Miller et al., 2014).

Two studies have identified that common genetic variants may also affect the lip region. One NSCL/P SNP near *IRF6* has been suggested to affect the relative protrusion of the lips in females (*P* = 6 × 10^−5^) (Peng et al., 2013), in addition, a candidate SNP near FGFR1 has showed nominal association with a long philtrum and wide mouth (*P* < 0.0005) and in the same study, a candidate SNP near LRP6 showed nominal association with a thick upper lip (*P* = 0.03) (Claes et al., 2014). A recent study has also demonstrated that the number of risk alleles that an individual has (polygenetic risk score) affects the width of the philtrum (Howe et al., 2018).

The lip region has subtle soft tissue variations, which are largely overlooked when using traditional facial land-marking methods. There is considerable variation in normal lip morphology, not only in terms of width and length, but also in terms of recognizable features such as lower lip drop, presence, or absence of lip borders and notches and grooves (Wilson et al., 2013). The aim of this study is to assess for associations between 19 independent NSCL/P SNPs and normal lip phenotypes.

## Methods

### Subjects

This study is based on three-dimensional facial data collected from 15-year-old children from the Avon Longitudinal Study of Parents and Children (ALSPAC; Boyd et al., 2013; Fraser et al., 2013). Ethical approval for the study was obtained from the ALSPAC Ethics and Law Committee and the Local Research Ethics Committees (UBHT): 06/Q2006/53^1^ Avon Longitudinal Study of Parents and Children (ALSPAC), Hands on Assessments: Teen Focus 3 (Focus 15+) (7th August 2006; Confirmed 15th September 2006). Written consent was also obtained from parents and guardians prior to obtaining the facial scans.

This prospective study recruited pregnant women living in the former county of Avon in South-West England with an estimated delivery date of between April 1st 1991 and December 31st 1992. The initial number of pregnancies enrolled was 14,541 (for these at least one questionnaire was returned or a “Children in Focus” clinic had been attended by 19/07/99). Of these initial pregnancies, there were a total of 14,676 fetuses, resulting in 14,062 live births and 13,988 children who were alive at 1 year of age. Please note that the study website contains details of all the data that is available through a fully searchable data dictionary and variable search tool^2^.

The children were invited to a research clinic when they were 15 years old. A subset of 4,747 children (2,233 males, 2,514 females) attended this clinic and had a three-dimensional facial scan taken using two Konica Minolta Vivid 900 laser cameras (Kau and Richmond, 2008, 2010). The reliability of image capture has been reported extensively elsewhere (Kau et al., 2005, 2006; Toma et al., 2009; Huang et al., 2011).

Each set of scanned images was imported into Rapidform 2006 (a reverse engineering software package). The color texture was removed and the facial shell was colored gray in order to highlight morphological features and eliminate the influence of differing facial color tones. The reverse engineering package enabled the full 360° rotation of the facial shell to identify surface contours.

Detailed assessment of lip morphology and surrounding area was conducted by CWN according to the Wilson-Richmond categorization scale (Wilson et al., 2013).

### Lip features

The Wilson-Richmond classification scale consists of 25 independent lip morphological features spanning from the philtrum to the sub-lip area and including the facial skeletal pattern (Table 1). A total of 118,675 (4747^*^25) phenotypes were captured.

**Table 1:** Description of morphological traits (accompanying illustrations in Supplementary Figure 1).

**Phenotype**		**Definition**	Type^*^
1	Philtrum shape	Progressive scoring of the surface of the philtrum in terms of the smoothness of the surface and the position of the largest indention from the columella to the vermilion border	M
2	Philtrum width	Three categories based on the width of the philtrum based anywhere from the columella to the vermilion border	O
3	Cupid's bow shape	Progressive scoring of the Cupid's bow the higher the score the more angulated the Cupid's bow	O
4	Nasolabial angle	Columella angle which can be acute, average or obtuse	O
5	Upper lip vermilion fullness	Progressive scoring of the fullness of the lip vermilion, not extending beyond the vermilion border (Viewed in profile)	O
6	Upper lip vermilion contour	The shape of the vermilion border from the Cupid's bow peaks to the commissures	O
7	Upper lip vermilion border	Identifiable vermilion lip border with variable coverage	B
8	Upper lip double vermilion border	A ribbon of soft tissue matching the vermilion border usually lying 2 mm above the border	B
9	Upper vermilion brim	A small semi-circular projection at the vermilion border	B
10	Upper lip vermilion midline groove	The presence of a midline groove: grooved area (tissue deficiency)	B
11	Upper lip vermilion midline drop	The presence of a midline drop: bumped area (tissue excess)	B
12	Lower lip vermilion fullness	Progressive scoring of the fullness of the lip vermilion, not extending beyond the vermilion border (Viewed in profile)	O
13	Lower lip vermilion contour	General curvature of the lower lip	O
14	Lower lip vermilion border	Identifiable vermilion lip border with variable coverage	B
15	Lower lip double vermilion border	A ribbon of soft tissue matching the vermilion border usually lying 2 mm below the border	B
16	Lower vermilion brim	A small semi-circular projection at the vermilion border	B
17	Lower lip vermilion midline groove	The presence of a midline grooved area (tissue deficiency)	B
18	Lower lip vermilion midline drop	The presence of a midline drop/bumped area (tissue excess)	B
19	Commissures	Position of the commissures in relation to the general lip line	O
20	Lower lip-chin shape	The curvature of the sub-lip area, from the lower lip vermilion border to the chin	O
21	Mentolabial fold	Presence of an obvious mentolabial fold	B
22	Chin dimple	Presence of an obvious chin dimple or cleft	B
23	Lower lip tone	The assessment of the mentolabial muscle tone viewed from the $\frac{3}{4}$ angle	O
24	Tone-up	The assessment of the mentolabial muscle tone viewed from the submentovertex (up) angle	O
25	Skeletal pattern	The clinical assessment of the relationship of the maxilla and mandible to the cranial base	O

* *Type denotes whether the variable is binary (B), ordinal (O) or multinomial (M)*.

### SNP selection and genotyping

ALSPAC children were genotyped using the Illumina HumanHap550 quad chip and imputed to 1000 Genomes (phase 1, version 3), as described in the Supplementary Material.

Nineteen independent SNPs (Table 2) were selected based on previously demonstrated association with clefting in European populations. The top SNP at each locus was selected. Subsequent SNPs identified at existing loci were assumed to not be independent unless this was demonstrated to be the case with conditional analysis. Dosages for the 19 NSCL/P SNPs were extracted from the imputed dataset. Allele dosages were flipped to correspond to the number of NSCL/P risk alleles at each SNP (allowing for non-integer values to account for uncertainty in the imputation). An un-weighted NSCL/P polygenetic risk score (PRS) was calculated as the sum of NSCL/P risk alleles for each individual (sum of risk allele dosages across all 19 loci). We were unable to generate a weighted score, which accounted for the relative effect sizes of the included SNPs, as it was not possible to extract equivalent effect size estimates for all the SNPs from the various GWAS publications.

**Table 2:** Candidate SNPs for NSCL/P identified by previous GWAS in European populations.

**SNP**	**Locus**	**Allele**	**Genomic location**	**Nearby candidate gene**	**Relative risk (95% CI)**		**Odds ratio (95% CI)**	**GWAS *P*-Values References**
					**RR het**	**RR hom**		
rs560426	1p21.3 1p22.1	**G**/A	Intronic	*ABCA4/ARHGAP29*	1.34 (1.13–1.60)	1.72 (1.38–2.14)		1.0 × 10^−6^ (Ludwig et al., 2012)
							1.43 (1.29–1.59)	5.0 × 10^−12^ (Beaty et al., 2010)
rs742071	1p36 1p36.1	**T**/G	Intronic	*PAX7*	1.25 (1.05–1.49)	1.80 (1.44–2.24)		2.6 × 10^−7^ (Ludwig et al., 2012)
							1.45 (1.26–1.67)	1.8 × 10^−5^ (Beaty et al., 2010)
							1.43 (1.24–1.66)	1.6 × 10^−6^ (Beaty et al., 2013)
rs861020	1q32.2	**A**/G	Intronic	*IRF6*	1.44 (1.22–1.69)	1.71 (1.24–2.35)		1.8 × 10^−6^ (Ludwig et al., 2012)
							1.43 (1.27-1.61)	1.2 × 10^−9^ (Beaty et al., 2010)
rs7590268	2p21	**G**/T	Intronic	*THADA*	1.42 (1.21–1.66)	2.04 (1.51–2.76)		1.3 × 10^−8^ (Ludwig et al., 2012)
					1.42 (1.26–1.59)	1.95 (1.56–2.44)		8.6 × 10^−8^ (Mangold et al., 2009)
rs7632427	3p11.1	C/**T**	Intergenic	*EPHA3*	0.79 (0.67–0.92)	0.63 (0.49–0.80)		4.2 × 10^−5^ (Ludwig et al., 2012)
rs793464	3q12.1	**A**/G	Intronic	*COL8A1/FILIP1L*			0.77 (0.68–0.88)	4.5 × 10^−5^ (Beaty et al., 2013)
rs79411602	6p21.3	A/**C**/T	Intergenic				1.52 (1.29–1.78)	2.9 × 10^−7^ (Leslie et al., 2016)
rs12543318	8q21.3	**C**/A	Intergenic	*DCAF4L2/MMP16*	1.26 (1.07–1.48)	1.83 (1.45–2.32)		1.0 × 10^−6^ (Ludwig et al., 2012)
rs987525	8q24	**A/**C	Intergenic	*MYC*	2.57 (2.02–3.26)	6.05 (3.88–9.43)		3.3 × 10^−24^ (Birnbaum et al., 2009)
							2.09 (1.59–2.76)	9.2 × 10^−8^ (Grant et al., 2009)
							1.78 (1.55–2.05)	1.1 × 10^−16^ (Beaty et al., 2010)
					2.07 (1.76–2.45)	4.68 (3.58–6.12)		3.9 × 10^−34^ (Ludwig et al., 2012)
rs1007966	9q22.1	A/**G**	Intergenic	*GADD45G*			1.29 (1.14–1.45)	3.0 × 10^−5^ (Beaty et al., 2013)
rs6478391	9q22.3	**C**/T	Intronic	*FOXE1*			1.19 (1.05–1.36)	6.8 × 10^−3^ (Beaty et al., 2013)
rs7078160	10q25	**A**/G	Intronic	*VAX1*	1.36 (1.21–1.53)	2.50 (1.95–3.21)		1.9 × 10^−8^ (Mangold et al., 2010)
							1.34 (1.20–1.50)	1.1 × 10^−7^ (Beaty et al., 2010)
					1.46 (1.24–1.72)	2.21 (1.56–3.15)		2.8 × 10^−8^ (Ludwig et al., 2012)
rs8001641	13q31.1	**A**/G	Intergenic	*SPRY2*	1.46 (1.20–1.78)	2.03 (1.62–2.56)		6.2 × 10^−10^ (Ludwig et al., 2012)
							1.86 (1.38–2.52)	4.0 × 10^−5^ (Jia et al., 2015)
rs1873147	15q22.2	**C**/T	Intergenic	*TPM1*	1.47 (1.25–1.72)	1.89 (1.44–2.47)		2.8 × 10^−8^ (Ludwig et al., 2012)
rs1880646	17p13.1	A/C/**T**	Intronic	*NTN1*			0.80 (0.72–0.88)	1.8 × 10^−5^ (Beaty et al., 2010)
							0.75 (0.66–0.86)	2.2 × 10^−5^ (Beaty et al., 2013)
rs227731	17q22	**C**/A	Intergenic	*NOG*	1.38 (1.21–1.56)	1.91 (1.63–2.24)		1.1 × 10^−8^ (Mangold et al., 2010)
								4.7 × 10^−5^ (Beaty et al., 2010)
					1.27 (1.07–1.52)	1.84 (1.48–2.28)		4.3 × 10^−8^ (Ludwig et al., 2012)
rs1588366	17q23.2	**A/**C/T	Intronic	*SOX9 /TANC2*			1.78 (1.46–2.17)	1.4 × 10^−8^ (Leslie et al., 2016)
rs2612753	17q25.3	A/C/G/**T**	Intronic	*RBFOX3*			1.25 (0.66–0.86)	3.3 × 10^−4^ (Beaty et al., 2013)
rs13041247	20q12	C/**T**	Intronic	*MAFB*			0.71 (0.64–0.78)	1.4 × 10^−11^ (Beaty et al., 2010)
					0.92 (0.79–1.08)	0.61 (0.47–0.78)		7.4 × 10^−4^ (Ludwig et al., 2012)

*Allele, minor allele is given first, with the risk allele shown in bold*.

*Betas, relative risks are given with the major allele set as baseline*.

*P-values all given for European populations (higher P values have been demonstrated with meta-analysis of multi-ethnic studies)*.

*Genes, The gene names listed are extracted from those reported in the original GWAS and often represent one of several nearby genes*.

### Statistical analysis

The PRS was tested for association with each of the 25 lip phenotypes. In addition, each SNP was also tested individually in a secondary analysis. Statistical analysis was performed using STATA software. Logistic, ordinal or multinomial regression analysis was performed according to whether the lip phenotype outcome was binary, ordered-categorical or unordered-categorical.

The strength of the evidence for association was assessed after accounting for multiple testing. For the association tests with the PRS, a Bonferroni correction was applied to account for the 25 phenotypes tested, α = 0.002 (0.05/25). In the association tests with individual SNPs, both the 25 phenotypes and the 19 SNPs were accounted for α = 1.1 × 10^−4^ (0.05/(25^*^19)).

## Results

The average NSCL/P PRS (indicating the average number of risk alleles carried) for the 3,643 ALSPAC individuals was 15.0 (95% CI 14.9-15.1), 15.0 in males and 14.9 in females. The scores varied from 6.0 to 24.5.

### Associations with NSCL/P PRS

Both philtrum width (*P* = 2 × 10^−4^) and Cupid's bow (*P* = 3 × 10^−4^) showed strong evidence (*p* < 0.002) for association with the NSCL/P PRS (Table 3). However the pseudo r^2^ estimates (0.0023 and 0.0021, respectively) suggest that the score only marginally improves model fit and the ORs (0.95 and 1.04, respectively) demonstrate modest effect sizes. The other traits tested showed little evidence for association with the PRS (Table 3, Supplementary Table 1).

**Table 3:** Logistic regression results of phenotypes associated with the NSCL/P PRS.

**Trait**	Odds Ratio^*^	**95% CI**	***P*-Value**	**Category associated with increased NSCL/P PRS**
Philtrum width	0.95	0.92–0.98	1.9 × 10^−4^	Narrow philtrum
Cupid's bow shape	1.04	1.02–1.07	2. × 10^−4^	V-shaped Cupid's bow
Commissure	0.98	0.96–1.00	0.07	
Tone up	1.02	0.99–1.04	0.12	
Upper lip groove	1.02	0.99–1.05	0.16	
Skeletal Pattern	0.98	0.96–1.01	0.18	
Philtrum Shape	NA +	NA+	0.20	
Lower lip Contour	1.01	0.99–1.04	0.22	
Dimple	0.98	0.96–1.01	0.25	
Lower lip drop	0.98	0.96–1.01	0.27	
Mentolabial fold	0.99	0.97–1.01	0.41	
Upper lip brim	0.99	0.96–1.02	0.43	
Upper lip border	0.99	0.95–1.02	0.48	
Nasolabial angle	1.00	0.98–1.03	0.58	
Upper lip contour	1.01	0.98–1.03	0.58	
Lip-chin shape	1.01	0.98–1.03	0.58	
Upper lip double border	1.01	0.96–1.07	0.60	
Lower lip brim	1.01	0.98–1.03	0.62	
Lower lip border	0.99	0.93–1.05	0.74	
Lower lip groove	1.00	0.97–1.04	0.79	
Upper lip drop	0.99	0.97–1.02	0.80	
Lower lip tone	1.00	0.98–1.03	0.81	
Lower lip double border	0.99	0.97–1.02	0.87	
Lower lip fullness	1.00	0.98–1.03	0.93	
Upper lip fullness	0.99	0.975–1.02	0.95	

**Figure 1:**
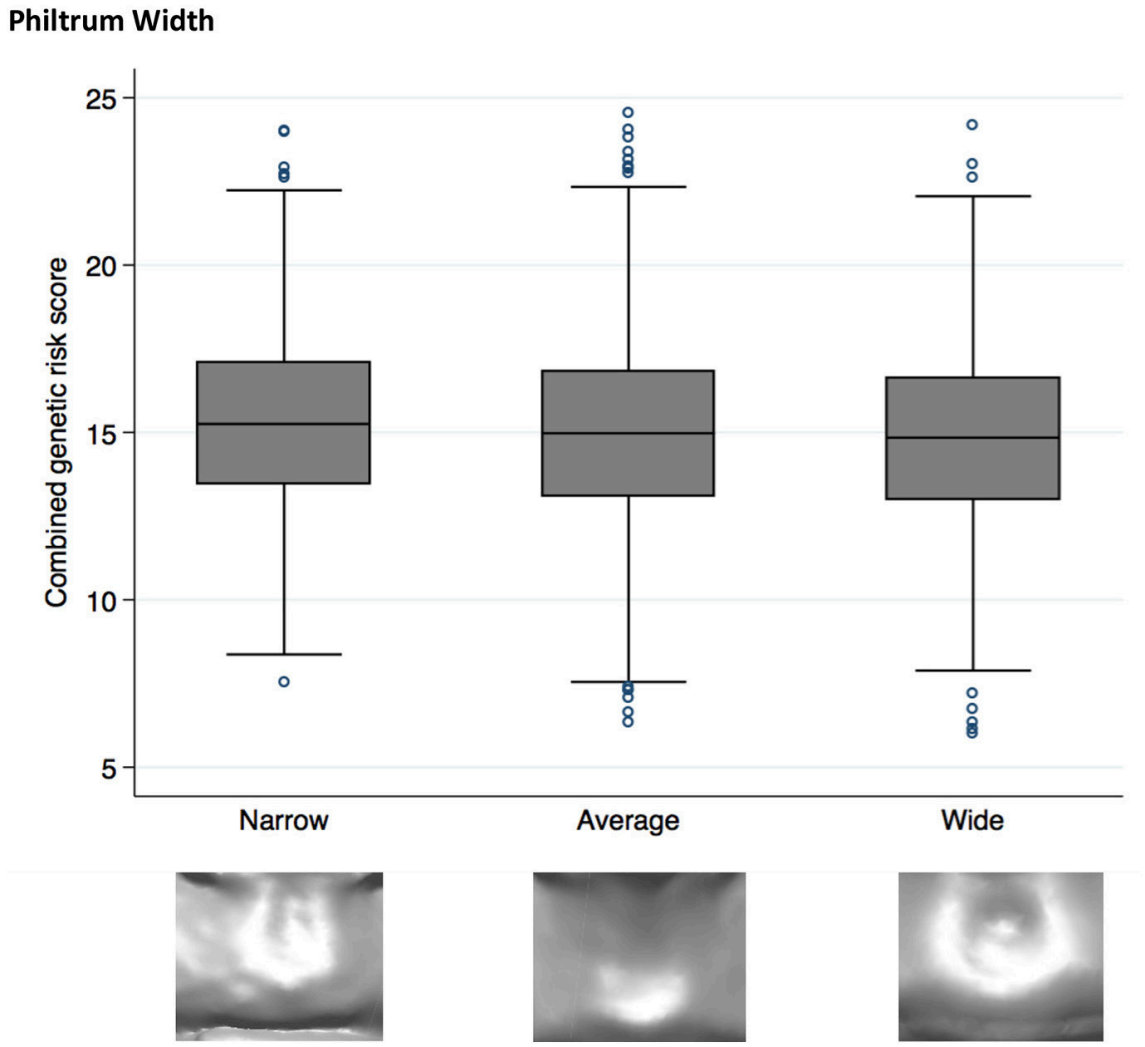
Box plot graph demonstrating the distribution of PRS for philtrum width phenotypes *P* = 1.9 × 10^−4^, OR 0.95 (0.92–0.98).

**Figure 2:**
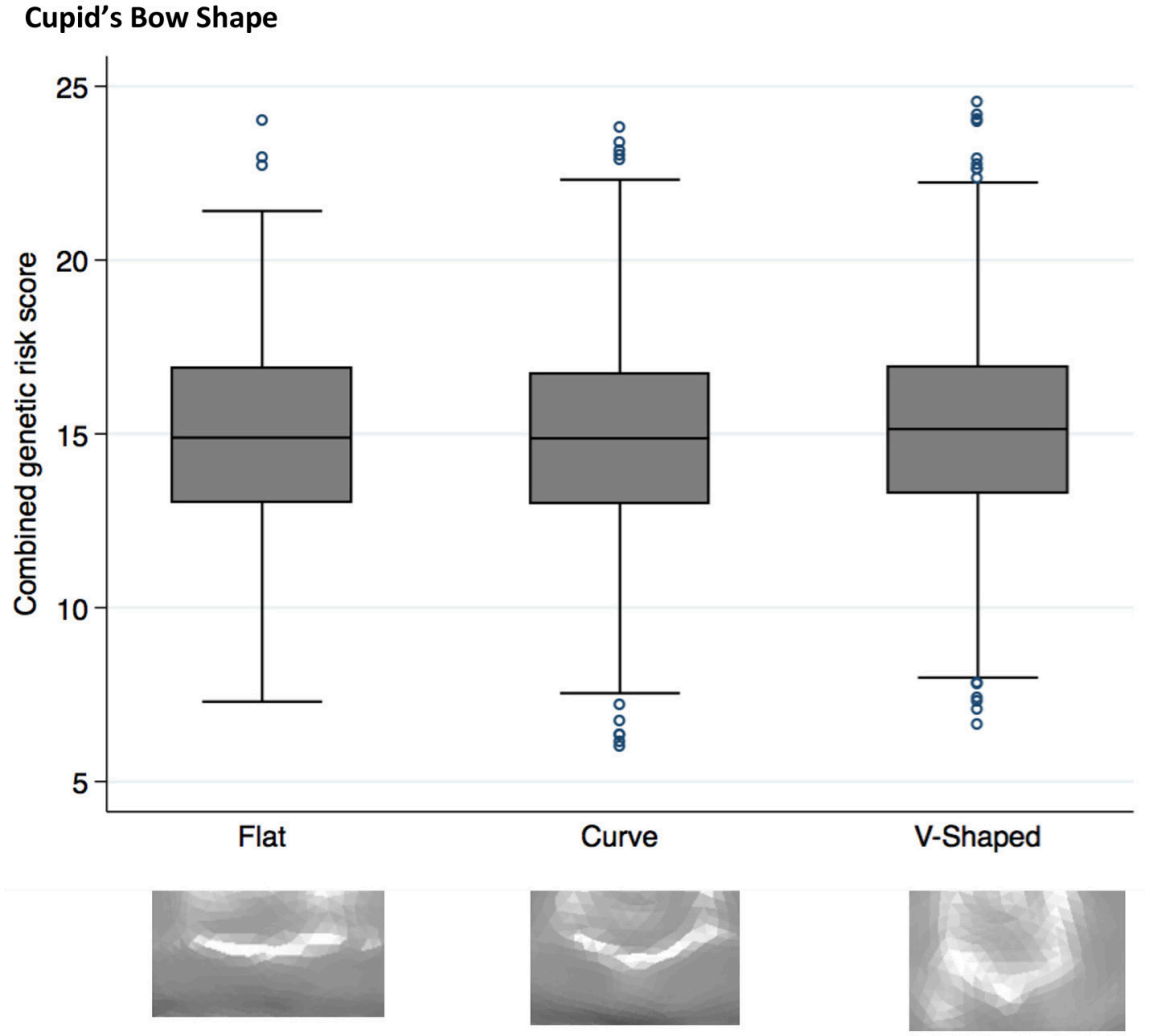
Box plot graph demonstrating the distribution of PRS for Cupid's bow shape phenotypes *P* = 2.6 × 10^−4^, OR 1.04 (1.02–1.07).

* OR are shown in units of per-allele increase in NSCL/P polygenic risk score. Both traits were analyzed as ordinal (according to the codes shown in Figures 1,2). To aid interpretation of these OR, in the far right column, for the two phenotypes which showed strong evidence for association, we have listed the phenotype with the greatest average NSCL/P polygenic risk score.

*+ The 6 multinomial ORs for philtrum shape are reported in Supplementary Table 1*.

Higher NSCL/P PRS was associated with a narrow philtrum (code 0, Figure 1) and V-shaped Cupid's bow (code 2, Figure 2). Individuals with a narrow philtrum had a higher average PRS (15.4) compared to those with a wide philtrum (14.7). A smaller difference was seen comparing those with a V-shaped (15.2) and a flat (14.9) Cupid's bow. Within this sample, 244 individuals had both a narrow philtrum and a V-shaped Cupid's bow and these had slightly increased PRS values of 15.6 compared to the total sample average of 15.0.

The 5% of individuals with the highest PRS (≥19.5) had a higher prevalence of V-shaped cupids bow (49%) and narrow philtrum width (16%), compared to the 5% of individuals with the lowest PRS (≤ 10.5), for whom prevalences were 33 and 8%, respectively. The prevalence of both features (narrow philtrum and a V-shaped Cupid's bow) together, was 13% amongst the 5% with the highest PRS compared to 3% amongst those with the lowest PRS.

### Multivariable regression of NSCLP SNPs and LIP phenotypes

#### Associations with individual NSCL/P SNPs

We investigated whether specific SNPs were driving the genetic risk for philtrum width and Cupid's bow shape. No SNPs showed strong evidence for association (*P* < 1.1 × 10^−4^) with these two traits, but for philtrum width, the strongest association was with rs7590268 [*P* = 3 × 10^−3^, OR 0.84 (0.74–0.94)]. For Cupid's bow shape, a suggestive association was also observed for the rs7590268 [*P* = 7 × 10^−3^, 1.16 (1.04 – 1.29)], in addition to rs987525 [*P* = 2 × 10^−3^, OR 1.18 1.06–1.32)] (Table 4). After adjusting for these individual nominally associated SNPs, the associations with the PRS remained associated for both traits (Cupid's bow shape *P* = 0.009, philtrum width *P* = 0.0012).

**Table 4:** Selection of NSCL/P SNPs nominally associated (*P* < 0.01) with lip phenotypes (results for all traits are in Supplementary Table 1).

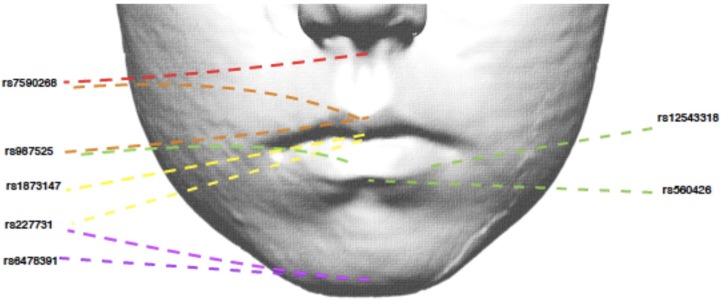								
**SNP**	**Risk Allele**	**Nearby candidate gene**	**Lip region**	OR^**^	**95% CI**	***P*-Value**	**Phenotype associated with NSCLP risk allele**	
rs227731	C	*NOG*	Skeletal	0.80	0.73–0.88	4.8 × 10^−6^^*^	0	Skeletal II
rs6478391	C	*FOXE1*	Skeletal	0.83	0.73–0.93	1.9 × 10^−3^	0	Skeletal II
rs7632427	T	*EPHA3*	Philtrum Shape	*1.01*	*0.81–1.26*	2.1 × 10^−3^	0	
				*0.90*	*0.72–1.11*		2	
				0.83	0.72–0.93		3	Indentation in middle (protective)
				*0.92*	*0.78–1.09*		4	
				*0.97*	*0.77–1.23*		5	
				0.64	0.51–0.81		6	Deep groove into vermilion (protective)
rs987525	A	*8q24*	Cupid's bow	1.19	1.06–1.32	2.3 × 10^−3^	2	V-shaped Cupid's bow
rs7590268	G	*THADA*	Philtrum width	0.84	0.74–0.94	2.5 × 10^−3^	0	Narrow philtrum width
rs560426	G	*ABCA4*	Lower lip drop	0.85	0.76–0.95	3.2 × 10^−3^	0	No lower lip drop
rs12543318	C	*DCAF4L2*	Lower lip contour	1.15	1.04–1.26	4.2 × 10^−3^	3	Markedly curved lower lip contour
rs227731	C	*NOG*	Upper lip drop	0.88	0.80–0.96	5.8 × 10^−3^	0	No upper lip drop
rs7590268	G	*THADA*	Cupid's bow	1.16	1.04–1.29	6.7 × 10^−3^	2	V-shaped Cupid's bow
rs1873147	C	*TPM1*	Upper lip groove	0.85	0.75–0.96	9.5 × 10^−3^	0	Absent upper lip groove

* SNP rs227731 and skeletal pattern II is the only association to meet the Bonferroni-corrected significance threshold (p < 1.2x10^−4^).

** *OR are shown in units of per-allele increase in NSCL/P risk. To aid interpretation of these OR, in the far right column we have listed the phenotype associated with the NSCL/P risk allele*.

**Figure 3:**
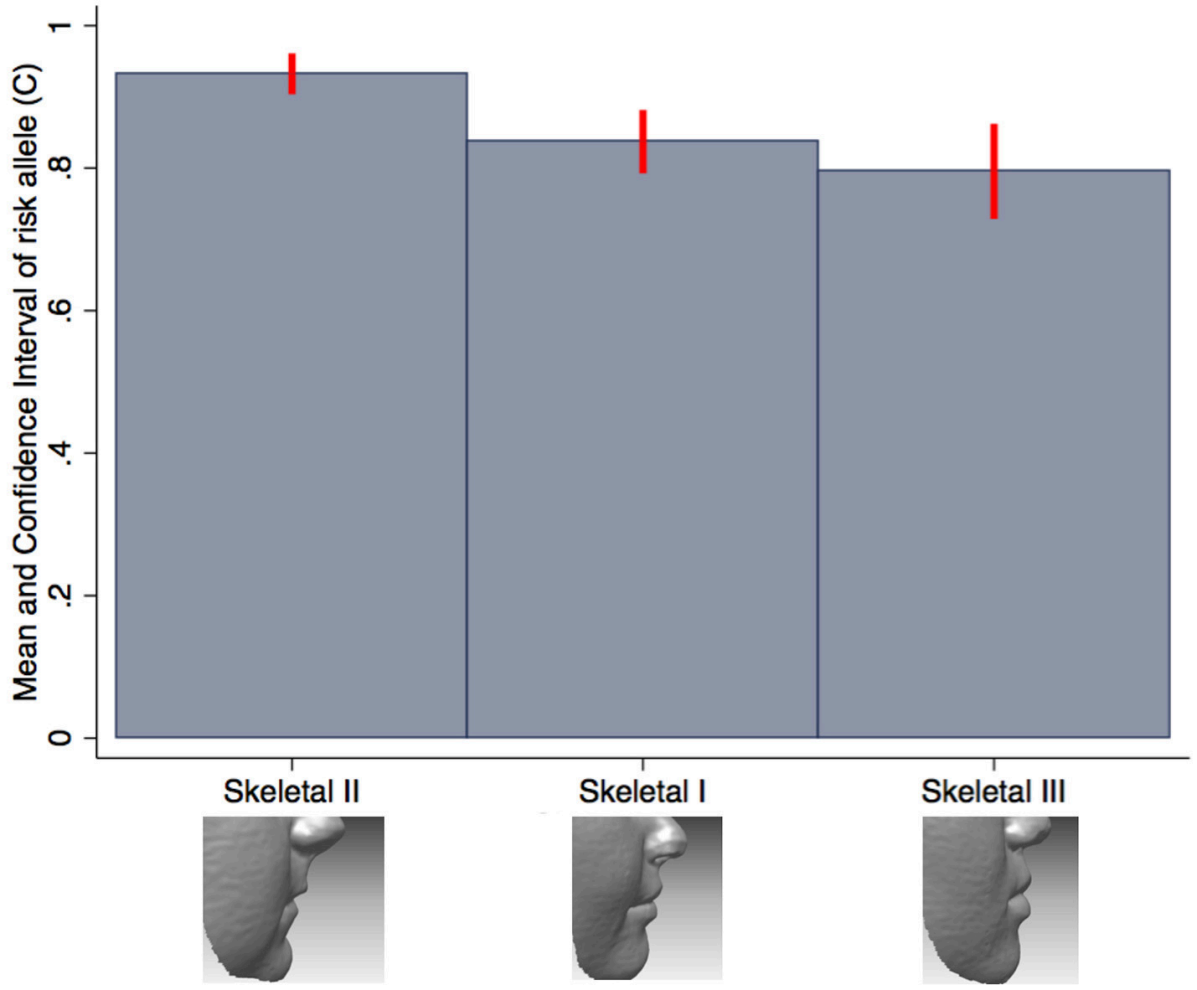
Bar chart of the mean number of rs227731 risk alleles, and confidence intervals according to the skeletal pattern. The only SNP to show strong evidence for association with a lip trait. A skeletal I describes a relationship whereby the maxilla and mandible are aligned in facial harmony, a skeletal II describes relative mandible retrognathia, and a skeletal III describes relative mandibular prognathia. A skeletal II pattern is associated with an increased number of rs227731 risk alleles [*P* = 5 × 10^−6^, OR 0.80 (0.73–0.88)].

We also tested the association between all SNPs and lip phenotypes that weren't strongly associated with the PRS. We found strong evidence (*P* < 1.1 × 10^−4^) for one association between rs227731 and skeletal pattern [*P* = 5 × 10^−6^, OR 0.80 (0.73–0.88, pseudo-*r*^2^ = 0.0033), where the NSCL/P risk allele (C) was associated with skeletal pattern II (retrognathic mandible; Figure 3). Figure 3 demonstrates that the average number of risk allele (C) decreases with a skeletal I (facial harmony), and further with a skeletal III pattern, suggesting that the cleft risk allele of rs227731 has a protective effect on prognathic mandible. We also found nominal association (*P* < 0.01) with seven other lip traits and individual SNPs (Table 4, Supplementary Table 1).

## Discussion

The nineteen genetic variants, which have been previously associated with NSCL/P in Europeans in genome-wide association studies (GWAS) appear to affect certain features of the lips and surrounding region. There appears to be evidence for an association between rs227731 and skeletal II pattern, and a PRS of the risk alleles shows an association with the philtral and Cupid's bow region.

### Skeletal pattern

SNP rs227731 and skeletal II pattern (relative mandibular retrognathia) was the only individual SNP analysis to show strong evidence for association [*P* = 4.8 × 10^−6^, OR 0.80 (0.73–0.88)]. This SNP is located 100 kb downstream of *NOG*, which encodes for the noggin protein and has been proposed as the likely candidate gene in this region (Mangold et al., 2010; Ludwig et al., 2012). There was little evidence for an association between the NSCL/P PRS and skeletal pattern (*P* = 0.18), indicating that the genetic overlap between these two phenotypes might be limited to this one locus, rather than skeletal pattern II being a general marker of high NSCL/P risk.

Research involving knockout mutant mice has demonstrated that mice lacking the *Nog* gene have enlarged mandibles (Matsui and Klingensmith, 2014). It is hypothesized that this manifests due to an increase in size of Meckel's cartilage during mandibular development, thus proposing that lack of *Nog* results in a prognathic mandible (skeletal III pattern) and an increase in *Nog* produces mandibular hypoplasia (skeletal II pattern). A genotype/phenotype relationship has been postulated between *Nog* and skeletal II pattern by several authors (Gutierrez et al., 2010; da Fontoura et al., 2015); however, this is the first study to demonstrate a potential association between this genomic region and skeletal pattern in humans.

### Philtrum

Although no individual SNP showed strong association with philtrum width, a narrow philtrum width was strongly associated with the increased NSCL/P PRS [*P* = 2 × 10^−4^, OR 0.95 (0.92–0.98)]. Individual SNP analysis revealed that rs7590268 was suggestively associated with philtrum width [*P* = 3 × 10^−3^, 0.84(0.75–0.94)]. The SNP rs7590268 was also associated with V-shaped Cupid's bow [*P* = 7 × 10^−3^, 1.16 (1.04–1.29)], and these two phenotypes are morphologically associated traits (*P* < 0.0005; Wilson-Nagrani, 2016). The proposed candidate gene for rs7590268 is *THADA* (Thyroid adenoma associated), although it has no known function in craniofacial development.

Philtrum shape was also nominally associated with SNP rs7632427 (*P* = 2.06 × 10^−3^), and indicated that the NSCL/P risk allele was protective for phenotypes “indentation in the middle” (Code 3) (OR 0.83 (0.72–0.93)) and “deep groove into the vermilion border” (Code 6) (OR 0.64 (0.51–0.81)). This SNP is located approximately 3 kb downstream of the *EPHA3* gene, this family of genes are involved in the regulation of cell shape and cell-cell contacts (Himanen et al., 2007).

A cleft lip and palate occurs within the philtrum and Cupid's bow region, and many theories relating to lack of migration, epithelial cell breakdown and lack of adhesion have been postulated (Mossey et al., 2009). The association of an increased PRS with narrow philtrum and V-shaped Cupid's bow seem highly plausible. Narrow philtrum and V-shaped Cupid's bow may exist as a result of lack of cell migration between the maxillary process and medial nasal processes, leading to reduced labial tissue. Reduction in philtrum width has also been associated with an increased PRS previously (Howe et al., 2018). The philtrum shapes of indentation in the middle and deep groove into the vermilion border show an apparent increase in labial tissue, and therefore this may contribute toward their protective role, in the reduction of risk alleles associated with SNP rs7632427.

### Cupids bow shape

A V-shaped Cupid's bow was strongly associated with an increased NSCL/P PRS (*P* = 3 × 10^−4^, OR 1.04 (1.02–1.07)). Two individual SNPs also show suggestive evidence for association with this phenotype; rs987525 (*P* = 2 × 10^−3^, 1.19 (1.06–1.32)).

The rs987525 SNP maps to an intergenic region of 8q24, which has been shown to control expression of the proto-oncogene Myc in the developing murine facial prominences (Uslu et al., 2014). This SNP was also found to be associated with bizygomatic distance in a GWAS study (Boehringer et al., 2011). Deletion of the Myc protein leads to mild alterations in facial morphology in mice, and sporadically leads to CL/P (Uslu et al., 2014).

### Upper lip

Two phenotypes of the upper lip were suggestively associated with individual SNPs. An absent upper lip drop was nominally associated with rs227731 [*P* = 6 × 10^−3^, 0.88(0.80–0.96)], and an absent upper lip groove with rs1873147 [*P* = 9 × 10^−3^, OR 0.85(0.75 – 0.96)].

An absent upper lip drop is an apparent reduction in lip tissue at the inferior border of the upper lip. SNP rs227731 has been described earlier in this article for its association with skeletal II pattern and its potential role in hypoplasia. An upper lip groove is a notched appearance to the inferior border of the upper lip, and is an apparent reduction in soft tissue at this site. An absent upper lip groove was nominally associated with rs1873147 [*P* = 9 × 10^−3^, OR 0.85 (0.75–0.96)].

### Lower lip

Two phenotypes of the lower lip were suggestively associated with NSCL/P SNPs. A lack of a lower lip drop was associated with rs560426 [*P* = 3 × 10^−3^, OR 0.85 (0.76–0.95)], this SNP has been previously associated with mouth width (Miller et al., 2014), a candidate gene at this locus, ARHGAP29, is expressed in the medial and lateral nasal processes, maxilla, mandible and secondary palatal shelves during craniofacial development (Leslie et al., 2012).

A markedly curved lower lip contour was also nominally associated with rs12543318 [*P* = 4 × 10^−3^, OR 1.15 (1.04–1.26)]. Rs12543318, lies within the 8q21.3 locus, whilst little is known about its function in craniofacial development, a few human chromosomal imbalances within this region have been found in patients with facial dysmorphology, including cleft lip (Beaty et al., 2010; Ludwig et al., 2012), and in particular cleft lip only (Moreno Uribe et al., 2017).

## Strengths and limitations

This is the first time that (at least some) established NSCL/P SNPs have been shown to have an effect on normal lip morphology. The use of a polygenic risk score (PRS) to investigate the genetic overlap between the traits is likely to increase power over the investigation of individual SNPs. We had 82% power to detect OR = 1.05 with the PRS (for a binary trait, α = 0.002, for the sample size of 3643, assuming a trait prevalence of 0.3). However, this power decreased to 53% for a trait prevalence of 0.1, or 7% power for an OR = 1.02 (calculated using G^*^Power 3.1.9.2). The power for the individual SNP analyses varied by allele frequency, but were a fraction of the power seen for the PRS analyses (for the same ORs), with 80% power only being achieved for example if the trait prevalence = 0.5 and OR = 1.26 (for risk allele frequency = 0.45). Therefore, though we found strong evidence for association for only one SNP-trait analysis and two score-trait analyses, we cannot rule out that there are weaker associations amongst the other variants and traits tested, due to the power limitations. Several additional analyses showed suggestive evidence of association and we would recommend that future studies attempt to replicate these findings.

Though we find evidence of associations, these represent small effects (as demonstrated by the small ORs and pseudo-r^2^ values). Therefore, although we have identified some interesting shared genetic effects between NSCL/P and normal lip morphology, these findings are unlikely to represent useful clinical predictors of NSCL/P.

Although additional loci have been associated with NSCL/P in the literature, we restricted our analysis to the 19 independent genetic variants, which have been associated with NSCL/P in genome-wide association studies (GWAS) of European populations, to best match the European ALSPAC population under study. Variants identified in other populations and not yet established in European populations may or may not be associated with NSCL/P in this population and further studies of Europeans will likely identify additional loci which when included in similar analyses will increase power. The variants we include come from GWAS analysis and are unlikely to represent causal variants, but this PRS approach to investigate shared genetic effects requires only proxy variants with strong association with NSCL/P and does not require the causal variants to be included. Along similar lines, our analysis can only claim to identify genetic loci of interest, and not the causal gene. However, for several of the loci studied, previous work has implicated specific candidate genes at the loci, which warrant further investigation.

A further limitation of this study is that we were unable to generate a weighted PRS (weighted by NSCL/P effect sizes), as it was not possible to extract equivalent effect size estimates for the SNPs from across the published GWAS studies. This was due to variations in study design (Case control or TDT) and some reporting relative risk or odds ratios, or not reporting European-only effect estimates. Whilst it would have been of interest to include a weighted score (and we hope that future publication of full summary statistics will make this possible), both un-weighted and weighted scores carry assumptions about the nature of associations, and it is not unreasonable to expect different relative effects of SNPs on different traits. Therefore, a score that summarizes only the number of risk alleles is still informative in investigating shared genetic effects.

## Conclusion

The aim of this study was to assess for associations between 19 NSCL/P SNPs and normal lip phenotypes. These subtle soft tissue variations have previously been overlooked when using traditional facial land-marking methods.

A PRS of the risk alleles suggests that NSCL/P SNPs may affect the morphology of the philtral area; eliciting a narrow philtrum and V-shaped Cupid's bow. These phenotypes may exist as a result of lack of cell migration between the maxillary process and medial nasal processes, leading to reduced labial tissue.

There was strong evidence for an association between rs227731 and skeletal II pattern, indicating relative mandibular retrognathia as the phenotype associated with the cleft risk allele at this locus, however, this phenotype does not appear to be associated with the PRS, and as such, is unlikely to be suggestive as a NSCL/P phenotype. A further eight of the nineteen SNPs reached nominal association (*p* < 0.01) with 10 lip phenotypes, and may warrant replication in future studies.

## Author contributions

CW-N performed the classification of the 4,747 children's lips according to the Wilson-Richmond categorization scale. CW-N and LP performed the statistical analysis. This study contributes toward CW-N's Ph.D. thesis (Wilson-Nagrani, 2016), and was supervised by SR.

### Conflict of interest statement

The authors declare that the research was conducted in the absence of any commercial or financial relationships that could be construed as a potential conflict of interest. The handling editor is currently collaborating with author SR, and confirms the absence of any other collaboration.

## Footnotes

^1^Ethical_approval_URL. from http://www.bristol.ac.uk/alspac/researchers/research-ethics/

^2^Data_Dictionary. from http://www.bristol.ac.uk/alspac/researchers/our-data/
