# Supplementary material for: Non-syndromic Cleft Lip and Palate Polymorphisms Affect Normal Lip Morphology
Source: Front Genet. 2018 Oct 24;9:413. doi: 10.3389/fgene.2018.00413 (PMC6207999; doi:10.3389/fgene.2018.00413)
Supplement: Supplementary file 2 [file Image_1.pdf]

	0	1	2	3	4	5	6
			**Philtrum**				
**Philtrum shape**	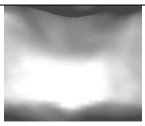 0.07	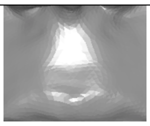 0.14	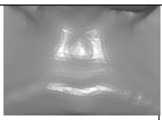 0.07	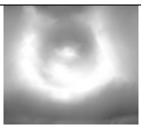 0.42	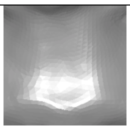 0.19	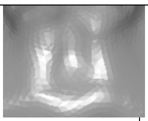 0.06	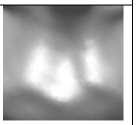 0.05
**Philtrum width**	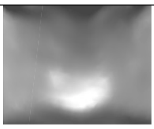 0.11	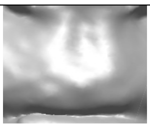 0.69	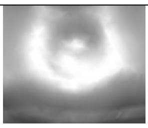 0.20				
**Cupid's bow shape**	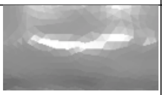 0.07	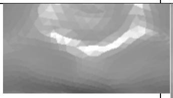 0.53	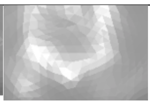 0.40				
**Nasolabial angle (Profile)**	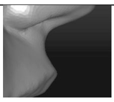 0.15	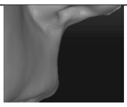 0.65	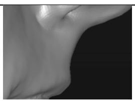 0.20				
			**Upper Vermilion**				
**Vermilion fullness (Profile)**	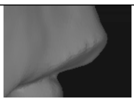 0.10	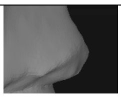 0.66	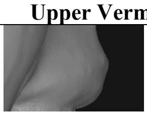 0.24				
**Vermilion contour**	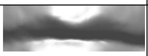 0.50	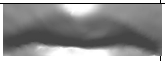 0.35	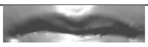 0.07	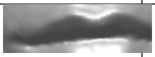 0.08			
**Vermilion border**	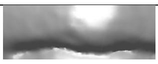 0.11	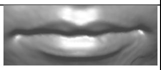 0.89					
**Double border**	0.94	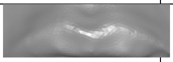 0.06					
**Vermilion brim (Profile)**	0.69	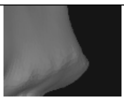 0.31					
**Vermilion groove**	0.76	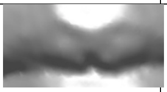 0.24					
**Vermilion drop**	0.41	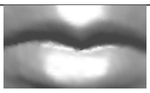 0.59					
			**Lower Vermilion**				
**Vermilion fullness (Profile)**	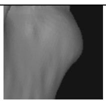 0.08	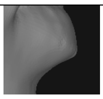 0.62	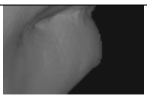 0.30				
**Vermilion contour**	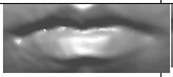 0.22	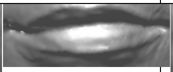 0.07	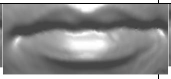 0.50	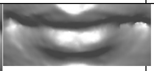 0.21			
**Vermilion border**	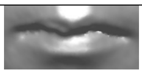 0.04	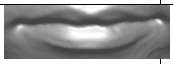 0.96					
**Double vermilion border**	0.56	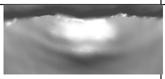 0.44					
**Vermilion brim (Profile)**	0.48	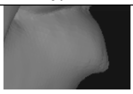 0.52					

**Vermilion groove**	**0.87**	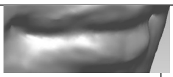 **0.13**					
**Vermilion bump**	**0.78**	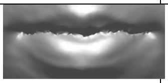 **0.22**					
			**Commissures**				
**Commissures**	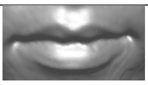 **0.10**	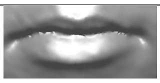 **0.37**	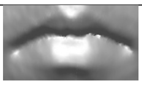 **0.53**				
			**Sub-Lip**				
**Lip-chin shape (Profile)**	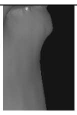 **0.04**	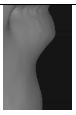 **0.37**	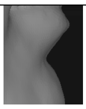 **0.39**	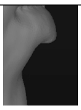 **0.12**	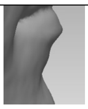 **0.08**		
**Mentolabial fold**	**0.56**	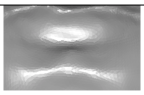 **0.44**					
**Chin dimple**	**0.78**	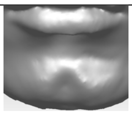 **0.22**					
**Lower lip tone (3/4 View)**	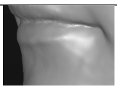 **0.07**	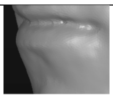 **0.13**	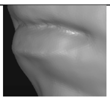 **0.36**	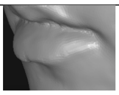 **0.34**	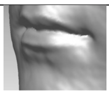 **0.10**		
**Lower lip tone (Looking up)**	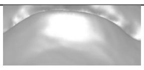 **0.17**	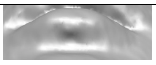 **0.13**	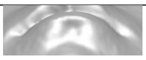 **0.29**	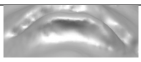 **0.33**	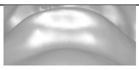 **0.09**		
**Skeletal pattern**	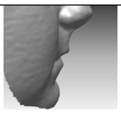 **0.25**	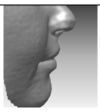 **0.64**	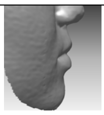 **0.11**				

Supplementary Figure 1 Classification scale for the characterisation of lip phenotypes and their proportions for the 4,747 ALSPAC population.
